# Post-tuberculosis tracheobronchial stenosis: long-term follow-up after self-expandable metallic stents placement and development of a prediction score—the Restenosis Score

**DOI:** 10.1186/s40001-022-00765-1

**Published:** 2022-07-27

**Authors:** Fuqi Li, Sen Tian, Haidong Huang, Wei Zhang, Yi Huang, Ning Wu, Qin Wang, Xiangqi Wang, Yuchao Dong, Chong Bai

**Affiliations:** 1 Department of Respiratory and Critical Care Medicine, Shanghai Changhai Hospital, The First Affiliated Hospital of Second Military Medical University, Shanghai, 200433 China; 2 Department of Pathology, Shanghai Changhai Hospital, The First Affiliated Hospital of Second Military Medical University, Shanghai, 200433 China

**Keywords:** Airway stenosis, Self-expandable metallic stents, Tuberculosis, Restenosis, Prediction

## Abstract

**Background:**

The insertion of self-expandable metallic stents (SEMS) for post-tuberculosis tracheobronchial stenosis (PTTS) was controversial. This study aimed to evaluate the efficacy and safety of SEMS for treating PTTS, and developed a scoring system for predicting the occurrence of restenosis after stenting in PTTS patients.

**Methods:**

We conducted a retrospective review of 87 patients who were diagnosed with PTTS and experienced SEMS insertion between January 2000 and December 2017. All procedures were performed via flexible bronchoscopy under conscious sedation and local anesthesia.

**Results:**

A total of 85 SEMS were successfully placed in 77 patients. Comparing with pre-stenting, there were significant improvements in the lumen diameters of the stenotic segment, mMRC scale and lung function after short-term SEMS placement. During the long-term (average 163.32 months) follow-up, 48 patients (62.3%) did not develop restenosis after stenting; the other 29 patients (37.7%) developed and eventually, 12 remained under interventional therapies and 11 had bronchial atresia. Multivariate Cox regression analysis revealed that the difference value between SEMS length and the stenosis-segment length, stenosis type, and the number of pre-stenting thermal ablation were independently related to restenosis occurrence and were subsequently used to establish the Restenosis Score. The model’s development group (0.83, 95% CI 0.74–0.92) and external validation set (0.94, 95% CI 0.77–1.00) showed excellent discrimination.

**Conclusion:**

SEMS placement could serve as a safe and effective treatment option for most patients with PTTS. Further, we built a prediction model depending on the independent predictors of restenosis occurrence, the Restenosis Score. This validated tool might provide a decision support and a better management for PTTS patients who underwent SEMS implantation.

## Introduction

Post-tuberculosis tracheobronchial stenosis (PTTS) is the leading cause of benign tracheobronchial stenosis (BTS) in the endemic areas of pulmonary tuberculosis [1]. The sleeve resection, as a frequently used surgical intervention, previously served as the gold standard of PTTS therapy [2, 3]. Currently, a minimally invasive therapeutic strategy—bronchoscopic procedure has been widely used to manage the disease, benefitting a proportion of selected cases who are not surgical candidates (i.e., multiple, long segments and poor pulmonary reserves). Typically, balloon dilation is the first treatment for PTTS, especially appropriate for annular cicatricial stenosis [4]. But when this approach fails and many a dilation is required, airway stenting is needed [4, 5].

Previous clinical evidences have indicated that silicone stents are a safe and effective treatment for patients with PTTS [6, 7]. Placement of silicone stents requires the additional rigid bronchoscopy, which promotes the development of self-expandable metallic stents (SEMS). SEMS are easily placed under local anesthesia by flexible bronchoscopy, avoiding the risk of perforation. From this perspective, SEMS have gained popularity and initial enthusiasm, with favorable short-term outcomes [8]. However, long-term follow-up presented unacceptable complication rates and difficulties of SEMS removal for patients with benign disease [9, 10]. This culminated in a public health warning against the use of SEMS in BTS according to the US Food and Drug Administration (FDA) in 2005 [11].

Despite the publishing of this advisory, the usage of SEMS in PTTS patients remained controversial and there were no studies elucidating the clinical predictors of restenosis occurrence. Hence, this retrospective study reported the clinical results of SEMS insertion in patients with PTTS and aimed to assess the safety and efficacy of this therapeutic modality. In addition, a scoring tool was developed and validated to predict the occurrence of restenosis for PTTS patients who experienced long-term SEMS placement.

## Materials and methods

### Patients

We retrospectively reviewed the medical records of all patients who received SEMS insertion for the treatment of PTTS at the First Affiliated Hospital of Second Military Medical University, Shanghai, from 1 January 2000 to 31 December 2008. The inclusion criteria were as follows: (1) time of SEMS implantation > 6 months; (2) location of stenosis is sole, that is, the stenosis only occurs in patients with left main bronchus, right main bronchus or bronchus intermedius. The exclusion criteria included: (1) absence of 6-month follow-up data after SEMS insertion; (2) stenosis of multiple locations. Given the retrospective nature that all data sources were based on the medical records, this study did not require ethics approval, which was renounced with a waiver of informed consent by the Institutional Review Board of the First Affiliated Hospital of Second Military Medical University, Shanghai.

### Stents

The types of SEMS used over the research included the uncovered SEMS (Nanjing Micro-Tech Co. Ltd., China) and the uncovered Ultraflex SEMS (Boston Scientific, USA).

### Airway intervention procedure

Airway stenosis was assessed using computed tomography (CT) and flexible bronchoscopy before the placement of SEMS. All procedures were conducted through flexible bronchoscopes (BF-1T260 and BF-C30 Olympus Corporation, Tokyo, Japan) under topical anesthesia and intravenous sedations. When stent removal procedure was needed, pre-procedure chest CT scans with flexible bronchoscopy were performed in all patients to thoroughly assess the condition of the airway, stent and vascular structures to the airway wall. This allowed for a detailed preoperative planning. The airway walls and stents were pretreated prior to removal with thermal ablation or cryotherapy in order to ablate the granulation tissue. If essential, balloon dilatation was performed to provide enough operation space for the operator. Next, the drawstring at the proximal or distal end of the stent was grasped with rigid alligator forceps which was inserted through working channel. The forceps was rotated and gentle, steady traction was then applied to withdraw the airway stent. The internal diameter at the stenotic segment was measured by chest CT before and after the procedure.

### Data collection

Baseline data obtained from the medical records included patient demographics, diagnosis, symptoms–diagnosis time window, location of airway stenosis, type of stenosis, previous treatments, type and length of SEMS, cause of SEMS replacement, type and number of other interventional bronchoscopy treatments, stent-related complications, and the luminal diameter of strictures. The symptoms–diagnosis time window was defined as the time from symptoms onset (e.g., dyspnea, chest discomfort, cough and hemoptysis) associated with endobronchial tuberculosis (EBTB) to diagnosis of EBTB. In combination with the classification of Freitag [12], there were 4 types of stenosis in this study: scarring, bronchomalacia, mixed (scarring and bronchomalacia) and bronchial atresia. The modified Medical Research Council (mMRC) Dyspnea Scale and lung function test were employed to evaluate the clinical outcomes before and after SEMS implantation. The follow-up was performed at 1 week, 1, 3, 6, and 12 months, and then annually until September 2019 after SEMS insertion, or when complications occurred or symptoms flared. In the light of the characteristics of SEMS-related complications, especially excessive granulation tissue formation, a joint management strategy of multiple airway intervention procedures (i.e., thermal ablation, cryotherapy and balloon dilation) was implemented to deal with symptom recurrence. If necessary, a new stent can be reinserted. The replacement of SEMS was dependent on the results of bronchoscopic follow-ups and long-term insertion of SEMS was defined as not less than 6 months without removal after stenting.

### Selection of predictor variables

A cross-sectional study was conducted for the predicted factors of restenosis occurrence in PTTS patients after SEMS placement. Predictors of restenosis considered in the model were easily measured and widely accepted in the clinical setting. The predictors selected by clinical reasoning aimed at minimizing noise and making the model easy to apply in clinical practice. Other predictors measured only by costly, time-consuming, or invasive procedures were not specifically considered.

### External validation

Data were collected from 10 PTTS patients experiencing the treatment of SEMS insertion at the First Affiliated Hospital of Second Military Medical University, Shanghai, between 1 January 2009 and 31 December 2017.

### Statistical analysis

All statistical analyses were carried out using SPSS Version 21.0 (IBM Corp, Chicago, IL, USA). Collected data were expressed as mean ± standard deviation (SD) or *n* (%). The luminal diameter of strictures, the mMRC Scale and data of lung function test pre- and post-stenting were compared by Student’s *t* test. The Chi-square (*χ*^2^) test was applied to the analyses of categorical variables and frequency percentages. The median time of restenosis after stenting was calculated by the Kaplan–Meier method. Univariate and multivariate analysis (using Cox regression model) were employed to ascertain the independent predictors of restenosis after 10-year stenting. The cut-off value was determined with acquiring the best Youden index that was defined as Sensitivity + Specificity − 1. The Restenosis Score prediction model was established depending on the results of multivariate Cox regression, and the verification of which was performed using ROC curve, Hosmer–Lemeshow goodness-of-fit test, *χ*^2^ test, log rank and Breslow test. *P* values < 0.05 were deemed to be statistically significant unless specified otherwise.

## Results

### Patients’ characteristics

**Fig. 1:**
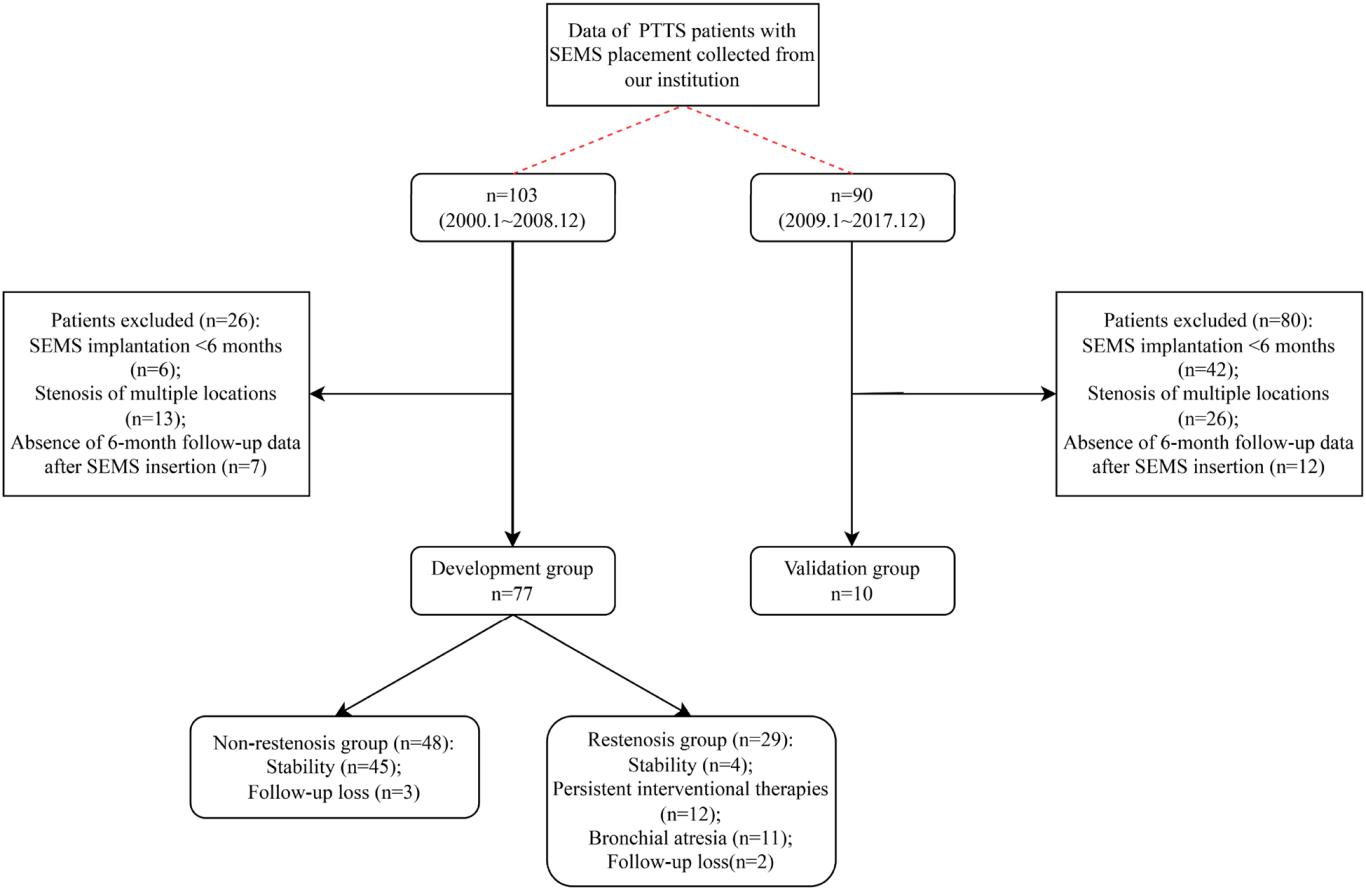
Flowchart of study population selections and outcomes

A total of 103 PTTS patients underwent SEMS insertion between January 2000 and December 2008 in our institution. According to the aforementioned inclusion criteria, 77 out of 103 (74.8%) PTTS patients were included in this study (Fig. 1). Table 1 shows patients’ baseline characteristics. In 77 enrolled patients, the median age was 32.77 ± 10.73 years and 75.3% were female. The symptoms–diagnosis time window was 5.62 ± 6.24 months. The mean duration of treat tuberculosis with anti-tubercular medications was 8.42 ± 5.30 months. All patients were diagnosed with airway stenosis related to EBTB. 85 SEMS were implanted in 77 patients with PTTS during the study period. 51 (60.0%) uncovered SEMS (Nanjing Micro-Tech Co. Ltd., China) and 34 (40.0%) uncovered Ultraflex SEMS (Boston Scientific, USA) were deployed successfully for PTTS patients involving left main bronchus (*n* = 65, 84.4%), right main bronchus (*n* = 7, 9.1%) and bronchus intermedius (*n* = 5, 6.5%).

**Table 1:** Baseline characteristics of the cohort

Characteristics	Total *N* = 77 (%)
Gender	
Male	19 (24.7)
Female	58 (75.3)
Age (range, years)	32.77 (16–57)
Symptoms–diagnosis time window (95%CI, months)	5.62 (4.20–7.04)
Anti-tuberculosis treatment	
Yes	75 (97.4)
No	2 (2.6)
Initial treatment or retreatment	
Initial treatment	66 (85.7)
Retreatment	11 (14.3)
Location	
Left main bronchus	65 (84.4)
Right main bronchus	7 (9.1)
Bronchus intermedius	5 (6.5)
Stent involved	85
SEMS (Nanjing Micro-Tech, China)	51 (60.0)
Ultraflex SEMS (Boston Scientific, USA)	34 (40.0)
Procedure method	
Flexible bronchoscopy	77 (100.0)
Rigid bronchoscopy	0 (0.0)
Anesthesia	
Local anesthesia	77 (100.0)
General anesthesia	0 (0.0)

### Short-term clinical outcomes and complications of SEMS

The cough, median mMRC scale and lumen diameters at the stenotic site for all patients 1 week after SEMS insertion (53.25%, 0.26 ± 0.05 and 8.23 ± 1.44, respectively) with no pending complications were significantly improved in comparison with those before SEMS insertion (32.47%, 1.60 ± 0.61 and 3.69 ± 1.35, respectively; cough: *P* = 0.009, mMRC scale: *P* < 0.001, lumen diameters: *P* < 0.001). Spirometry tests showed statistically significant increases in the mean values of forced expiratory volume in 1 s (FEV1), forced vital capacity (FVC), FEV1 predicted and FVC predicted from baseline. Six patients with atelectasis of left lung resulting from complete occlusion of the left main bronchus experienced almost totally successful recruitment after 1-week SEMS placement. Compared with pre-stenting, there were statistically significant improvements in the pectoralgia, mean mMRC scale and lumen diameters of the stenotic segment after 6-month stenting. Furthermore, a statistical difference in the palliation of the cough was observed between 1 week and 6 months after stenting (*P* = 0.04) (Table 2).

**Table 2:** The improvements of respiratory status after short-term period of SEMS placement

Respiratory status	Pre-stenting	1 week after stenting (*P* value)	6 months after stenting (*P* value)
Lumen diameter (mean ± SD, mm)	3.69 ± 1.35	8.23 ± 1.44 (< 0.001)	6.49 ± 1.54 (< 0.001)
Spirometry tests			
FEV_1_ (mean ± SD, L)	2.09 ± 0.55	2.44 ± 0.67 (< 0.001)	NA
FEV_1_ (% predicted, mean ± SD)	67.04 ± 15.07	79.25 ± 19.52 (< 0.001)	NA
FVC (mean ± SD, L)	2.53 ± 0.61	2.91 ± 0.72 (< 0.001)	NA
FVC (% predicted, mean ± SD)	67.45 ± 13.68	76.96 ± 16.95 (< 0.001)	NA
FEV_1_/FVC (mean ± SD)	82.39 ± 8.72	83.56 ± 8.91 (0.199)	NA
Symptom			
mMRC scale (mean ± SD)	1.60 ± 0.61	0.26 ± 0.05 (< 0.001)	0.13 ± 0.34 (< 0.001)
Cough (%)	25 (32.5)	41 (53.2) (0.009)	28 (36.4) (0.61)
Pectoralgia (%)	23 (30.0)	32 (41.6) (0.13)	13 (16.9) (0.03)

*FEV*_*1*_ forced expiratory volume in one second; *FVC* forced vital capacity; *mMRC* modified Medical Research Council; *NA*, not applicable

During the period of 6-month follow-up, a total of 23.4% (18 out of 77) patients developed the stent-related complications, including granulation proliferation (*n* = 15, 19.5%), overgrowth of necrotic tissue (*n* = 1, 1.3%), and migration (*n* = 2, 2.6%). SEMS replacement occurred in 8 patients because of the inappropriate size of stents (*n* = 6) and stent migration (*n* = 2). All 8 patients underwent a successful removal of the SEMS without significant complications. In addition, 11 patients suffered restenosis due to overgrowth of granulation tissue in the 1st (*n* = 2), 2nd (*n* = 1), 3rd (*n* = 2), 4th (*n* = 3) and 5th (*n* = 3) months.

### Long-term clinical outcomes and complications of SEMS

At the average follow-up duration of 163.32 months, 48 patients (62.3%) did not develop restenosis after SEMS insertion, the other 29 patients (37.7%) developed. The results suggested that the type of stenosis of restenosis patients was of statistical difference with non-restenosis patients (*P* = 0.042). For restenosis patients, mixed stenosis (*n* = 13, 44.8%) and cicatricial stenosis (*n* = 10, 34.5) were the major types of stenosis. Moreover, restenosis patients presented better epithelialization of SEMS by comparison of non-restenosis patients, with a statistical significance (*P* = 0.003). However, there was no significant difference in age, sex, site of stenosis, the number of other interventional treatments before stenting, and the type of SEMS between restenosis and non-restenosis patients (Table 3).

**Table 3:** Baseline characteristics of the restenosis and non-restenosis group

Characteristics	Restenosis group (*N* = 29)	Non-restenosis group (*N* = 48)
Gender		
Male (%)	7 (24.1)	12 (25.0)
Female (%)	22 (75.9)	36 (75.0)
Age (mean ± SD, years)	30.31 ± 8.99	34.25 ± 11.49
Location		
Left main bronchus (%)	26 (89.7)	39 (81.3)
Right main bronchus (%)	2 (6.9)	5 (10.4)
Bronchus intermedius (%)	1 (3.4)	4 (8.3)
Stent involved		
SEMS (Nanjing Micro-Tech, China) (%)	19 (65.5)	26 (54.2)
Ultraflex SEMS (Boston Scientific, USA) (%)	10 (34.5)	22 (45.8)
Stenosis type		
Scarring (%)	10 (34.5)	20 (41.7)
Bronchomalacia (%)	2 (6.9)	13 (27.1)
Mixed (%)	13 (44.8)	13 (27.1)
Bronchial atresia (%)	4 (13.8)	2 (4.1)
Number of pre-stenting interventions	164	179
Thermal ablation	34 (20.7)	26 (14.5)
Balloon dilation	124 (75.6)	153 (85.5)
Cryotherapy	6 (3.7)	0 (0.0)
Median duration of implantation (mean ± SD, months)	132.38 ± 64.05	164.17 ± 44.00
Neo-epithelialization (%)	16 (55.2)	41 (85.4)
Complications		
Granulation tissue (%)	26 (89.7)	23 (47.9)
Scarring tissue (%)	15 (51.7)	3 (6.3)
Sputum retention (%)	3 (10.3)	2 (4.2)
Necrotic tissue (%)	0 (0.0)	1 (2.1)
Stent breakage (%)	0 (0.0)	2 (4.2)
Infection (%)	0 (0.0)	1 (2.1)
Outcomes		
Stability (%)	4 (13.8)	45 (93.8)
Still under treatment (%)	12 (41.4)	0 (0.0)
Bronchial atresia (%)	11 (37.9)	0 (0.0)
Surgery (%)	9 (81.8)	0 (0.0)
Lost follow-up (%)	2 (6.9)	3 (6.2)

**Fig. 2:**
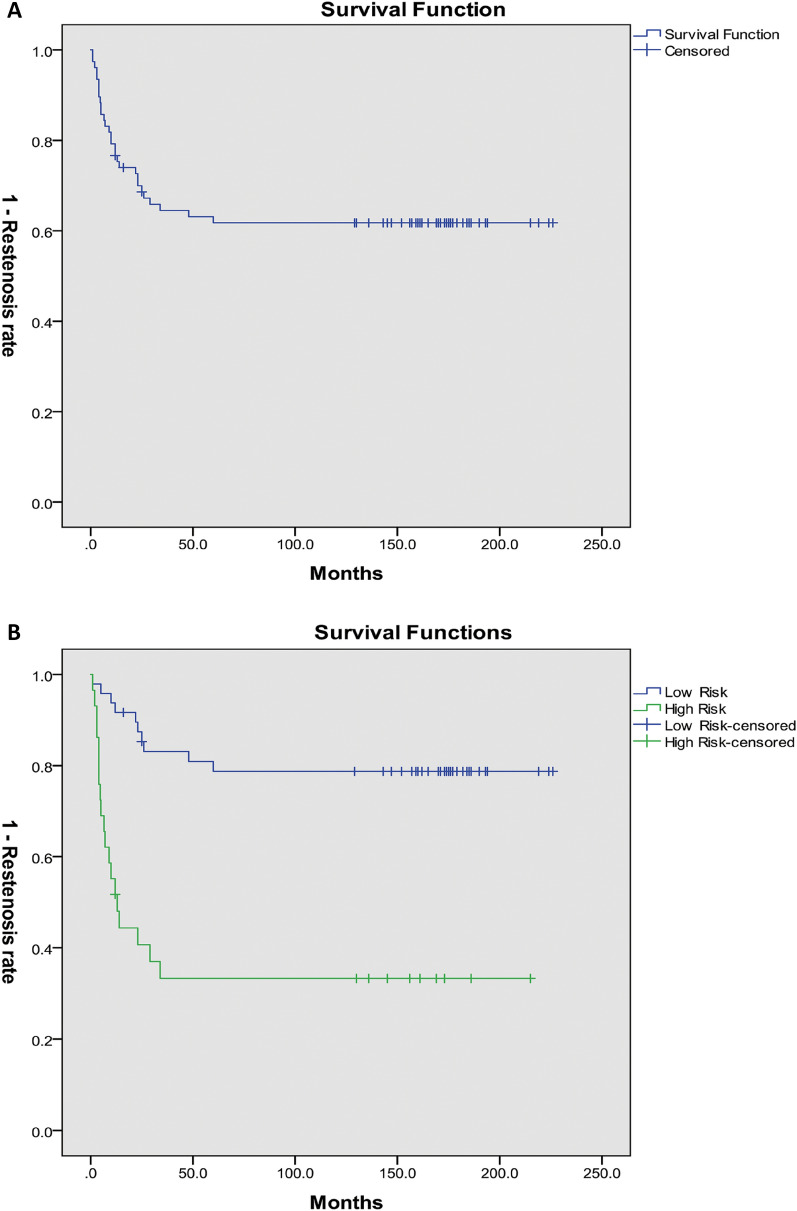
Kaplan–Meier survival curve of restenosis: **A** all patients; **B** low-risk and high-risk patients

During the stenting period of 152.19 ± 54.31 months, almost all patients experienced stent-related late complications in which granulation tissue formation (63.6%) and scarring tissue proliferation (23.4%) commonly occurs. Furthermore, the rate of overgrowth of granulation tissue resulting in restenosis reached 33.8%. Figure 2A shows the Kaplan–Meier survival curve of restenosis after SEMS placement. The 1-month, 6-month, 1-year, 3-year and 5-year restenosis rates were 2.6%, 14.3%, 23.4%, 35.1% and 37.7%, respectively. But the median time of restenosis after stenting was not estimated by this curve.

#### Restenosis group

Among 29 restenosis patients, the median duration of restenosis was 10 months (range from 1 to 60 months). Granulation tissue ingrowth (90.0%), scarring tissue proliferation (51.7%) and secretion retention (10.3%) were three common complications of SEMS (Table 3). A variety of repeat interventional treatments had been performed to deal with the aforementioned complications. During at least 10 years of follow-up, 4 (13.8%) patients displayed a stable clinical condition, 12 (41.4%) required persistent interventional therapies, 11 (37.9%) had bronchial atresia and 9 of them were referred for surgery, and 2 (6.9%) were lost to follow-up. The mean duration of SEMS implantation in restenosis group was 132.38 ± 64.05 months (Fig. 1). With respect to prognosis, the cough (37.0%) and median mMRC scale (1.78 ± 1.01) were not significantly improved compared with pre-stenting (24.1% and 1.66 ± 0.61, respectively; cough: *P* = 0.224, mMRC scale: *P* = 0.43). The worse shortness of breath occurred in 2 patients with poor response to stenting, severely affecting quality of life. In all restenosis patients, significant improvements in the pectoralgia and lung function were observed at 10 years after stenting (Table 4).

**Table 4:** The improvements of respiratory status after long-term period of SEMS placement

Respiratory status	Restenosis group			Non-restenosis group		
	Pre-stenting	10 years after stenting	*P* value	Pre-stenting	10 years after stenting	*P* value
Lumen diameter (mean ± SD, mm)	NA	NA	NA	3.51 ± 1.42	6.29 ± 1.10	< 0.001
Spirometry tests						
FEV_1_ (mean ± SD, L)	2.08 ± 0.57	2.42 ± 0.44	< 0.001	2.09 ± 0.54	2.68 ± 0.58	< 0.001
FEV_1_ (% predicted, mean ± SD)	65.51 ± 13.00	76.48 ± 7.14	< 0.001	69.83 ± 13.10	88.33 ± 7.96	< 0.001
FVC (mean ± SD, L)	2.51 ± 0.68	3.02 ± 0.59	< 0.001	2.55 ± 0.57	3.29 ± 0.71	< 0.001
FVC (% predicted, mean ± SD)	63.51 ± 11.69	77.11 ± 6.06	< 0.001	69.83 ± 14.34	88.24 ± 8.08	< 0.001
Symptom						
mMRC scale (mean ± SD)	1.66 ± 0. 61	1.78 ± 1. 01	0.43	1.56 ± 0.62	0.07 ± 0.25	< 0.001
Cough (%)	7 (24.1)	10 (34.5)	0.224	18 (37.5)	10 (20.8)	0.084
Pectoralgia (%)	7 (24.1)	1 (3.4)	0.033	16 (33.3)	0 (0.0)	< 0.001

*FEV1* forced expiratory volume in one second; *FVC* forced vital capacity; *mMRC* modified Medical Research Council; *NA* not applicable

#### Non-restenosis group

At the stenting duration of 164.17 ± 44.00 months, the overall incidence of stent-related complications was 66.7% (32/48), including granulation tissue formation (47.9%), scarring tissue proliferation (6.3%), necrotic tissue overgrowth (2.1%), mucus plugging (4.2%), stent fracture (4.2%), and infections (2.1%) (Table 3). These were of minor severity, and were self-correcting or appropriately managed with observation or endoscopic intervention. During the period of follow-up, 93.8% (45 out of 48) patients exhibited a stable clinical condition in which no severe complication developed due to the well-tolerated SEMS. The other 3 patients were lost to follow-up (Fig. 1). With regard to prognosis, the median luminal diameter of the stenotic segment increased from 3.51 ± 1.42 mm to 6.29 ± 1.10 mm (*P* < 0.001). The functional effects of which were embodied as significant improvements on spirometry, the pectoralgia and the mMRC Scale. However, no significant improvement in the cough was examined between pre-stenting and 10 years after stenting (*P* = 0.084) (Table 4).

### Restenosis Score prediction model

#### Univariate and multivariate Cox regression analysis

A total of 13 variables which were selected in this cross-sectional study were bound up with restenosis in patients after stenting and readily available in the clinical setting. These variables consisted of sex, age, symptoms–diagnosis time window, pre-stenting anti-tubercular therapy, initial treatment, type of stenosis, the number of pre-stenting interventional treatments (i.e., thermal ablation, cryotherapy and balloon dilation), type of SEMS, the difference value of the luminal diameter pre- and post-stenting, the difference value between the external diameter of SEMS and the luminal diameter before stenting, and the difference value between the length of SEMS and the length of the stenosis segment. Univariate Cox regression analysis indicated that the statistical effects of the difference value between the length of SEMS and the length of the stenosis segment, the number of pre-stenting thermal ablation and cryotherapy were significant. Type of stenosis did not reached a statistical significance (*P* = 0.09), however, it was included in the followed multivariate Cox regression analysis on the basis of clinical evidences and published articles [13, 14]. On the contrary, considering small sample size in the training cohort, the number of pre-stenting cryotherapy was excluded.

#### The construction of Restenosis Score prediction model

**Fig. 3:**
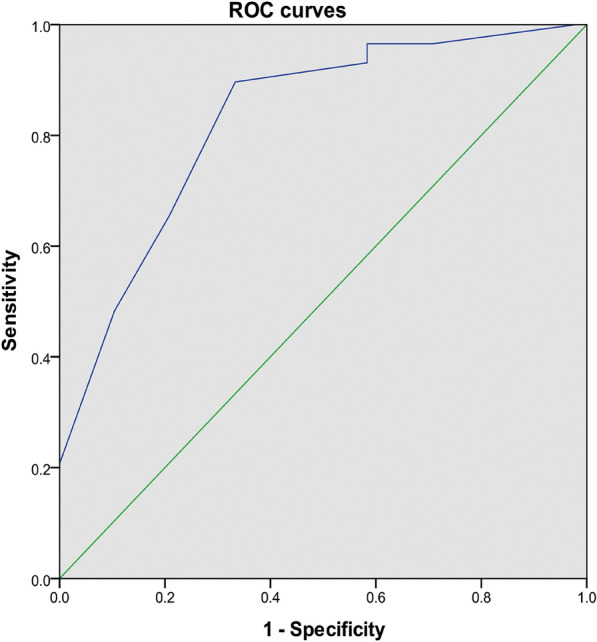
ROC curve of the development group

After final multivariate Cox regression analysis, the Restenosis Score retained type of stenosis, the difference value between the length of SEMS and the length of the stenosis segment, and the number of pre-stenting thermal ablation (Table 5). A detailed description of the Restenosis Score is also displayed in Table 5. The Restenosis Score of all patients is from − 8 to 8, and higher score is interrelated to greater predicted incidence of restenosis. The area under the receiver-operating characteristic curve (AUROC) in the development group was 0.83 (95% CI 0.74–0.92, *P* < 0.001), implying that there was a significant discrimination with the Restenosis Score prediction model (Fig. 3). Hosmer–Lemeshow *χ*^2^ of 5.8 (*P* = 0.33) in the development group signified good model calibration. The development group and validation group were separated into two risk stratification depending on the cut-off point:  ≤ 0 (low risk),  > 0 (high risk). The restenosis rates of the development group in low-risk and high-risk patients were 20.8% and 65.5%, with a statistical significance (*P* < 0.001). There were significant differences in the Kaplan–Meier restenosis survival curve between low-risk and high-risk patients after stenting (log rank or Breslow test, *P* < 0.001). The median time of restenosis with high-risk patients was 13.00 ± 3.44 months, however, low-risk patients failed to be estimated (Fig. 2B).

**Table 5:** Results of multivariate Cox regression analysis and Restenosis Score

Variables	*P* value	HR	95% CI	*β*	Points
Stenosis type					
Scarring	0.01	1			0
Bronchomalacia		0.58	0.12–2.73	− 0.28	− 1
Mixed		4.03	1.55–10.51	1.44	3
Bronchial atresia		1.44	0.39–5.43	0.39	1
Number of pre-stenting thermal ablation					
0	0.001	1	1.44–8.07		0
1–2		3.41	2.43–36.15	1.26	3
≥ 3		9.36	1.44–8.07	2.28	5
Difference value between SEMS length and stenosis-segment length (cm)					
≤ 0	0.002	1			0
0.01–1		0.30	0.12–0.74	− 1.28	− 3
> 1		0.12	0.03–0.42	− 2.19	− 4

*HR* hazard ratio; *CI* confidence interval; *SEMS* self-expandable metallic stents

#### Verification of model performance

We then employed the external validation group to verify the above findings from the development group. The external validation group comprising 10 patients demonstrated a restenosis rate of 80%. ROC analysis utilizing the Restenosis Score displayed an excellent discrimination with an AUC of 0.94 (95% CI 0.77–1.00) and the Hosmer–Lemeshow analysis for the Restenosis Score showed good calibration (*χ*^2^ = 3.29, *P* = 0.771). Applying the aforesaid risk classification to the external validation cohort yielded a restenosis rate of 33.3% for low-risk (*n* = 3), and 100.0% for high-risk (*n* = 7) patients, respectively. The difference of restenosis rate between low-risk and high-risk patients reached a marginal statistical significance (Fisher’s exact test, *P* = 0.067).

## Discussion

To our best knowledge, the present study is the largest experience to date reporting on the safety and efficacy of SEMS in PTTS. Besides, we performed the first study that developed a scoring tool to predict the occurrence of restenosis for PTTS patients after long-term placement of SEMS.

In this study, there was a prominent preponderance of women (75.3%), as previously observed [7, 15, 16]. A prospective study was conducted by Jung [17], who even incorporated female gender into an independent predictor of concomitant endobronchial tuberculosis. This phenomenon may be because; women do not normally expectorate sputum due to sociocultural and esthetic factors [18], and have narrower bronchus than men [19], which results in a longer exposure to tubercle bacilli, thus makes women more susceptible to endobronchial tuberculosis. Clearly, more data and further studies are needed.

Compared to silicone stents, SEMS exhibit theoretical advantages such as the self-expansible property, the ease of placement and so on. Nevertheless, the high complication rates of SEMS insertion in BTS should not be ignored. In the series conducted by Dooms [20], the short-term (< 12 weeks) complication rate after stenting was 75%, requiring stent removal in 60%. Their team therefore forsook the usage of SEMS for BTS patients in their clinical practice. This is completely inconsistent with the outcomes of our study in that almost all patients presented clinical improvements with low short-term (< 6 months after SEMS deployment) complication rate (31.7%) and all stent-related complications could be properly and successfully managed under endoscopy. This inconsistency may be explained by accumulation of experience. We must realize that experience with the use of SEMS is of great clinical importance since it directly affects the prognosis of BTS patients who undergo SEMS implantation.

The short-term safety and efficacy of SEMS placement in PTTS have been extensively documented in the present and previous studies [21–23]. Nevertheless, the long-term benefit of which still remains to be uncertain. Recently, there has been some research showing positive outcomes. Zhou evaluated the long-term results of temporary placement of SEMS in 40 BTS patients [21]. They reported a 2–4 weeks recurrence rate of 45.0%. A retrospective review conducted by Jeong [24] showed the clinical outcomes of complications following SEMS implantation for BTS. The incidence of restenosis was found to be 42.9% during a median follow-up period of 40 months. By comparison, the study of Fortin reported less recurrence rate (30.8%) in 13 BTS patients with an elective stent removal trial after a median dwell time of 223.5 ± 95.8 days [22]. Similarly, it was revealed in the study of Kim [23], the recurrence rate of 6-month stenting group was significantly lower than that of 2-month stenting group (41.7% vs 83.3%, *P* = 0.045). In addition, Chen [25] suggested that the optimal duration of stent placement was 4–8 months. In our study, the median 152.19 ± 54.31 months’ stenting could give rise to less recurrence rate than that in the 6-month stenting group in the study of Kim [23]. The lower recurrence rate might be due to the dilation effect of SEMS that could afford an opportunity for the stenosis site to remodel or heal.

**Fig. 4:**
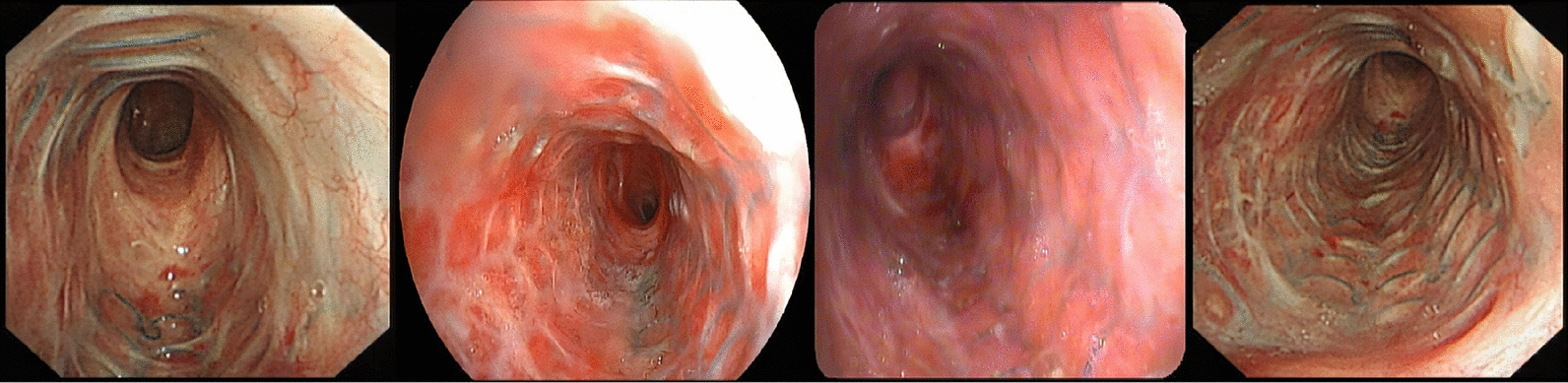
Bronchoscopy images of metal stent neo-epithelialization from 4 different patients

Further, our study indicated that non-restenosis group displayed decreased complication rates compared with restenosis group (granulation tissue proliferation, 47.9% vs. 89.7%; scar hyperplasia, 6.3% vs. 51.7%; mucostasis, 4.2% vs. 10.3%, respectively). These differences may attribute to the fact that non-restenosis group have the significant advantage of epithelialization with incorporation of the stent into the airway wall relative to restenosis group (85.2% vs. 55.2%, *P* = 0.003), which is instrumental in normal mucociliary clearance of secretion [26]. It was noted that neo-epithelialization within the stent was related to the type of SEMS, and uncovered SEMS preferred the theoretical benefit of neo-epithelialization [27, 28]. According to our study, for patients with good epithelialization within the stent (SEMS is closely connected with the airway mucosa) (Fig. 4), permanent placement of SEMS could be considered. However, granulation tissue that produces recurrent obstruction inside the stent could also lead to the neo-epithelialization of the stent, making its removal extremely difficult and requiring repeated debridement [29].

Contrary to the aforementioned studies in which placing the SEMS longer means better long-term benefit, there were a multitude of reports regarding the high incidence of complications after SEMS insertion with a longer period [24, 30, 31]. In our study during a median follow-up period of 163.32 months, the incidence of overgrowth of necrotic tissue, stent fracture, mucostasis and infection were significantly decreased in comparison with those reported in other research [14, 21, 22, 24, 30]. Furthermore, there was a relatively higher incidence of granulation tissue proliferation (63.6%) than those reported in published studies with a frequency of 14.6–47.8% [9, 14, 21, 31, 32], which might be interpreted by longer follow-up duration and larger size of patients, and could be acceptable when taking the successful endoscopic management of most complications into account. Hopefully, identifying the etiopathogenesis of granulation tissue hyperplasia and the technical improvements in SEMS design would contribute to better management in PTTS patients who underwent SEMS insertion. With respect to stent removal, there have been several studies reporting stent removal success rates of 84.5–98.2% with a rigid bronchoscopy and high complication incidence during or after stent removal [24, 32, 33]. In the current study, 8 patients needed SEMS replacement due to the inappropriate size of stents (*n* = 6) and stent migration (*n* = 2). They all underwent a successful removal of the SEMS without any complications, which was to be unexpected since these stents were uncovered and could become embedded into the mucosal wall. This difference may be attributable to small quantity of patients and the management strategy with skilled teams.

Relatively poor prognosis in restenosis group raises a question that what the independent predictors of restenosis occurrence are for PTTS patients who experienced SEMS placement. Our results of multivariate Cox regression analysis showed that the difference value between the length of SEMS and the length of the stenosis segment might be the protective factor, the number of pre-stenting thermal ablation and type of stenosis served as the risk factors. The Restenosis Score prediction model was subsequently developed on a basis of the aforesaid results, and the performance of which was further enhanced by a validation group which was somewhat small (only 13.0% of the development group). Such a small sample of validation cohort could be closely associated with the restraint (for the use of SEMS in BTS) which was issued by the FDA in 2005 [11]. Since then, just a few cases received this therapeutic modality. Given the range of the 95% CI, the validation group may lack of adequate power to predict the occurrence of restenosis. Nevertheless, the results of the external date validation should be considered acceptable when put into the small quantity of validation group.

Based on our Restenosis Score prediction model, there are several suggestions when placing the SEMS into PTTS patients. First, patients with bronchomalacia stenosis are better suited for SEMS implantation than those with scarring stenosis. As previously proven, symptoms of airway obstruction in patients with bronchomalacia stenosis could be alternatively relieved with a lower incidence of obstructive granulomas [14]. Second, a satisfactory sizing of SEMS should surpass the full length of stenosis, which is in line with that of silicone stents [34]. Third, before stenting, patients are recommended to avoid thermal ablation and receive tailored interventional therapies, such as cryotherapy and balloon dilation.

The present study has several limitations. Although this is the largest series to date associated with SEMS in PTTS, the sample size (77 patients) of this single-center study with the retrospective nature is relatively small especially external validation group (10 patients), which leads to be not representative of the whole population of PTTS patients with SEMS insertion. Hence, applying these results to other institutions should be cautious and further multicenter studies with larger sample size are needed to verify our findings. In addition, we have not been completely elucidated the independent predictors of restenosis occurrence due to inadequate data and small sample size, other possible independent predictors, such as the type and number of post-stenting nebulized therapy, and the genetic differences of hyperplastic granulation tissue remain to be explored. In particular, we highlight that our Restenosis Score, although validated and useful, serves only as a supplementary tool to facilitate decisions that experienced interventional pulmonologists make after weighting the benefit and risk of SEMS placement.

## Conclusions

Taken together, our analysis indicates that SEMS placement is safe and effective for all the patients with PTTS in a short-term follow-up and for most of those in a long-term follow-up. Further, we develop a simple and validated tool—the Restenosis Score, which can predict the occurrence of restenosis for PTTS patients after SEMS insertion and be easily utilized in clinical practice. We anticipate it will provide a decision support for physicians when considering insert SEMS into PTTS patients.

## Data Availability

All the data from this manuscript are publically available.
